# Supplementary material for: Bioinformatics and Experimental Analyses Reveal Immune-Related LncRNA–mRNA Pair AC011483.1-CCR7 as a Biomarker and Therapeutic Target for Ischemic Cardiomyopathy
Source: Int J Mol Sci. 2022 Oct 9;23(19):11994. doi: 10.3390/ijms231911994 (PMC9569729; doi:10.3390/ijms231911994)
Supplement: Supplementary file 1 [file ijms-23-11994-s001.zip › Supplementary Tables.pdf]

Table S2 Immune-related mRNAs

SYMBOL	ENSEMBL
A2M	ENSG00000175899
ACTA1	ENSG00000143632
AGTR1	ENSG00000144891
AIMP1	ENSG00000164022
AKT3	ENSG00000117020
AKT3	ENSG00000117020
AMHR2	ENSG00000135409
AMHR2	ENSG00000135409
APOM	ENSG00000204444
BIRC5	ENSG00000089685
BPIFB1	ENSG00000125999
BPIFB3	ENSG00000186190
BPIFB4	ENSG00000186191
BPIFC	ENSG00000184459
BRD8	ENSG00000112983
C3	ENSG00000125730
C3	ENSG00000125730
CAT	ENSG00000121691
CBL	ENSG00000110395
CCL21	ENSG00000137077
CCL21	ENSG00000137077
CCL21	ENSG00000137077
CCR7	ENSG00000126353
CCR7	ENSG00000126353
CCR7	ENSG00000126353
CD209	ENSG00000090659
CD8A	ENSG00000153563
CD8A	ENSG00000153563
CD8A	ENSG00000153563
CD8B	ENSG00000172116
CD8B	ENSG00000172116
CHGB	ENSG00000089199
CHUK	ENSG00000213341
CHUK	ENSG00000213341
CMTM5	ENSG00000166091
CRHR2	ENSG00000106113
CRLF3	ENSG00000176390
CSF1R	ENSG00000182578
ELAVL1	ENSG00000066044
F2R	ENSG00000181104
FGF1	ENSG00000113578
FGFR3	ENSG00000068078
FLT4	ENSG00000037280
FYN	ENSG00000010810
FYN	ENSG00000010810
GALP	ENSG00000197487
GHRHR	ENSG00000106128
GIPR	ENSG00000010310
GLP1R	ENSG00000112164
GMFG	ENSG00000130755
GZMB	ENSG00000100453

HLA-DMB	ENSG00000242574
HLA-DOB	ENSG00000241106
HNF4A	ENSG00000101076
HSPA2	ENSG00000126803
HTR3C	ENSG00000178084
IFIH1	ENSG00000115267
IFITM1	ENSG00000185885
IGHG2	ENSG00000211893
IGKC	ENSG00000211592
IL10	ENSG00000136634
IL10	ENSG00000136634
IL10	ENSG00000136634
IL11RA	ENSG00000137070
IL11RA	ENSG00000137070
IL16	ENSG00000172349
IL16	ENSG00000172349
IL17D	ENSG00000172458
IL17D	ENSG00000172458
IL17RB	ENSG00000056736
IL17RB	ENSG00000056736
IL18RAP	ENSG00000115607
IL18RAP	ENSG00000115607
IL22RA1	ENSG00000142677
IL22RA1	ENSG00000142677
IL24	ENSG00000162892
IL24	ENSG00000162892
IL6R	ENSG00000160712
IL6R	ENSG00000160712
IL7R	ENSG00000168685
IL7R	ENSG00000168685
IL7R	ENSG00000168685
IRF1	ENSG00000125347
IRF7	ENSG00000185507
ITGB2	ENSG00000160255
ITK	ENSG00000113263
JAG2	ENSG00000184916
KL	ENSG00000133116
LGR6	ENSG00000133067
MICB	ENSG00000204516
MICB	ENSG00000204516
MPO	ENSG00000005381
MR1	ENSG00000153029
MUC4	ENSG00000145113
NCR3	ENSG00000204475
NFYC	ENSG00000066136
NLRX1	ENSG00000160703
NR1D2	ENSG00000174738
NR1H2	ENSG00000131408
NRG4	ENSG00000169752
OAS1	ENSG00000089127
OASL	ENSG00000135114
OSGIN1	ENSG00000140961
PAEP	ENSG00000122133

PAK1	ENSG00000149269
PAK1	ENSG00000149269
PAK2	ENSG00000180370
PDK1	ENSG00000152256
PGLYRP4	ENSG00000163218
PLXNA2	ENSG00000076356
PLXNA2	ENSG00000076356
PLXNC1	ENSG00000136040
PLXNC1	ENSG00000136040
PPBP	ENSG00000163736
PPBP	ENSG00000163736
PPBP	ENSG00000163736
PPIA	ENSG00000196262
PRLHR	ENSG00000119973
PTGFR	ENSG00000122420
PTK2B	ENSG00000120899
PTK2B	ENSG00000120899
RABEP2	ENSG00000177548
RARA	ENSG00000131759
RASGRP3	ENSG00000152689
RHOA	ENSG00000067560
S100A1	ENSG00000160678
S100A14	ENSG00000189334
SEMA3G	ENSG00000010319
SEMA3G	ENSG00000010319
SEMA4B	ENSG00000185033
SEMA4B	ENSG00000185033
SEMA4C	ENSG00000168758
SEMA4C	ENSG00000168758
SHC2	ENSG00000129946
SKIV2L	ENSG00000204351
SLIT1	ENSG00000187122
SLIT1	ENSG00000187122
SORT1	ENSG00000134243
SP1	ENSG00000185591
SSTR2	ENSG00000180616
STC2	ENSG00000113739
TEK	ENSG00000120156
TGFA	ENSG00000163235
TGFBR1	ENSG00000106799
TGFBR1	ENSG00000106799
TGFBR2	ENSG00000163513
TGFBR2	ENSG00000163513
TIE1	ENSG00000066056
TNFRSF12A	ENSG00000006327
TNFRSF12A	ENSG00000006327
TNFRSF17	ENSG00000048462
TNFRSF17	ENSG00000048462
TPM2	ENSG00000198467
TRAV12-3	ENSG00000211794
TSLP	ENSG00000145777
TXLNA	ENSG00000084652
TXLNA	ENSG00000084652

UBR1	ENSG00000159459
VCAM1	ENSG00000162692
WFIKKN1	ENSG00000127578
ZC3HAV1	ENSG00000105939

Table S3 Immune-related lncRNAs

SYMBOL	ENSEMBL
LINC01409	ENSG00000237491
AC117945.1	ENSG00000234481
MYCL-AS1	ENSG00000236546
AL355483.1	ENSG00000228838
AC099063.4	ENSG00000284734
LINC01750	ENSG00000231437
AL049825.1	ENSG00000285698
FP700111.1	ENSG00000224363
UBE2Q1-AS1	ENSG00000229780
AL365181.2	ENSG00000272068
AL590666.2	ENSG00000229953
AL139011.1	ENSG00000228606
AL356441.1	ENSG00000236206
LINC00862	ENSG00000203721
LINC01460	ENSG00000205334
AL133243.3	ENSG00000276517
MAP4K3-DT	ENSG00000231312
AC016727.1	ENSG00000270820
AC016727.3	ENSG00000285857
AC018462.1	ENSG00000229839
AC007277.1	ENSG00000213981
AC007405.3	ENSG00000235934
SGO1-AS1	ENSG00000231304
AC006059.5	ENSG00000287629
KIF9-AS1	ENSG00000227398
SEMA3F-AS1	ENSG00000235016
GLYCTK-AS1	ENSG00000242797
AC007126.1	ENSG00000250497
KLF3-AS1	ENSG00000231160
AC097478.4	ENSG00000288563
H2AZ1-DT	ENSG00000245322
SCOC-AS1	ENSG00000196951
AC079768.2	ENSG00000287298
AC011407.1	ENSG00000287814
LINC01951	ENSG00000204754
ZSCAN16-AS1	ENSG00000269293
LINC02536	ENSG00000281248
AL592429.2	ENSG00000226571
AL358852.1	ENSG00000278899
NANOGP11	ENSG00000233464
AL606970.1	ENSG00000224417
AL109924.4	ENSG00000285917
AL109924.2	ENSG00000234519
SNHG26	ENSG00000228649
AC093582.1	ENSG00000236529

LINC00174	ENSG00000179406
AC002451.1	ENSG00000231170
AC010973.2	ENSG00000244151
PRKAG2-AS1	ENSG00000239911
AC022239.2	ENSG00000286985
AC079193.2	ENSG00000249258
AP006248.4	ENSG00000279932
AC027117.1	ENSG00000253671
AC100802.1	ENSG00000253775
AC015687.1	ENSG00000285579
AL360020.1	ENSG00000286834
LINC00702	ENSG00000233117
AL353576.1	ENSG00000287925
STAM-AS1	ENSG00000260589
NUTM2B-AS1	ENSG00000225484
ENTPD1-AS1	ENSG00000226688
AC023282.1	ENSG00000274461
WDR11-AS1	ENSG00000227165
LINC00958	ENSG00000251381
AC104009.1	ENSG00000254768
LRP4-AS1	ENSG00000247675
AP002336.2	ENSG00000254604
AC004803.1	ENSG00000250132
AC008124.1	ENSG00000273015
AC148477.4	ENSG00000277011
AL442125.1	ENSG00000276248
LINC00452	ENSG00000229373
AL136419.2	ENSG00000285727
AL137230.2	ENSG00000258792
LINC00605	ENSG00000251533
AC090510.4	ENSG00000286877
AC025271.4	ENSG00000276772
AC103739.3	ENSG00000285560
AL032819.1	ENSG00000260132
LINC01569	ENSG00000262468
AC138811.1	ENSG00000260017
AC138028.6	ENSG00000278341
AC026254.2	ENSG00000266313
AC022903.2	ENSG00000285559
NBR2	ENSG00000198496
AC007114.2	ENSG00000263089
AC015813.1	ENSG00000264112
AC007922.3	ENSG00000265094
AC005776.2	ENSG00000273218
AC011483.1	ENSG00000267879
AC006115.2	ENSG00000286125
A1BG-AS1	ENSG00000268895
SLCO4A1-AS1	ENSG00000232803
BX322562.1	ENSG00000273796
AL022318.5	ENSG00000288106
AL096840.3	ENSG00000287384
AL445253.1	ENSG00000285873
LINC02574	ENSG00000233975

LINC01788	ENSG00000229051
AL513285.1	ENSG00000226208
AL121987.1	ENSG00000225279
AL353593.2	ENSG00000270110
AC074117.1	ENSG00000234072
AC069404.1	ENSG00000287435
C3orf35	ENSG00000198590
Z84492.1	ENSG00000271858
FAM153CP	ENSG00000204677
HCG25	ENSG00000232940
LINC00513	ENSG00000233559
AF235103.1	ENSG00000255164
ZNF252P-AS1	ENSG00000255559
PITRM1-AS1	ENSG00000237399
AL391684.1	ENSG00000224934
KCNQ1DN	ENSG00000237941
AP003721.2	ENSG00000256733
AP003068.2	ENSG00000254614
AP003498.1	ENSG00000248671
LINC00368	ENSG00000225870
PWRN4	ENSG00000260232
AC093525.7	ENSG00000269937
AC093525.4	ENSG00000261140
AC108134.3	ENSG00000262370
AC135050.6	ENSG00000278133
AC087164.1	ENSG00000266677
CCDC144NL-AS1	ENSG00000233098
AC099811.3	ENSG00000267658
HOXB-AS2	ENSG00000239552
AC087741.1	ENSG00000262580
AC008764.2	ENSG00000268087
LIPE-AS1	ENSG00000213904
AC021092.1	ENSG00000186019
AC016629.2	ENSG00000269600
AL035071.1	ENSG00000260257
AL499627.1	ENSG00000260542
AL357033.1	ENSG00000167046
LINC01651	ENSG00000279920
EFCAB6-AS1	ENSG00000223843

Table S5 GSE116250 sample information

Group	GEO Accession	Organism	Platform ID	Disease state	Tissue
NF1	GSM3219558	Homo sapiens	GPL16791	Non-failing	heart
NF2	GSM3219565	Homo sapiens	GPL16791	Non-failing	heart
NF3	GSM3219566	Homo sapiens	GPL16791	Non-failing	heart
NF4	GSM3219567	Homo sapiens	GPL16791	Non-failing	heart
NF5	GSM3219568	Homo sapiens	GPL16791	Non-failing	heart
NF6	GSM3219569	Homo sapiens	GPL16791	Non-failing	heart
NF7	GSM3219570	Homo sapiens	GPL16791	Non-failing	heart
NF8	GSM3219571	Homo sapiens	GPL16791	Non-failing	heart
NF9	GSM3219559	Homo sapiens	GPL16791	Non-failing	heart
NF10	GSM3219560	Homo sapiens	GPL16791	Non-failing	heart
NF11	GSM3219561	Homo sapiens	GPL16791	Non-failing	heart

NF12	GSM3219562	Homo sapiens	GPL16791	Non-failing	heart
NF13	GSM3219563	Homo sapiens	GPL16791	Non-failing	heart
NF14	GSM3219564	Homo sapiens	GPL16791	Non-failing	heart
ICM1	GSM3219609	Homo sapiens	GPL16791	ischemic	heart
ICM2	GSM3219610	Homo sapiens	GPL16791	ischemic	heart
ICM3	GSM3219611	Homo sapiens	GPL16791	ischemic	heart
ICM4	GSM3219612	Homo sapiens	GPL16791	ischemic	heart
ICM5	GSM3219613	Homo sapiens	GPL16791	ischemic	heart
ICM6	GSM3219614	Homo sapiens	GPL16791	ischemic	heart
ICM7	GSM3219615	Homo sapiens	GPL16791	ischemic	heart
ICM8	GSM3219616	Homo sapiens	GPL16791	ischemic	heart
ICM9	GSM3219617	Homo sapiens	GPL16791	ischemic	heart
ICM10	GSM3219618	Homo sapiens	GPL16791	ischemic	heart
ICM11	GSM3219619	Homo sapiens	GPL16791	ischemic	heart
ICM12	GSM3219620	Homo sapiens	GPL16791	ischemic	heart
ICM13	GSM3219621	Homo sapiens	GPL16791	ischemic	heart

Table S6 GSE46224 sample information

Group	GEO Accession	Organism	Platform ID	Disease state	Tissue
NF1	GSM1126612	Homo sapiens	GPL11154	Non-failing	heart
NF2	GSM1126613	Homo sapiens	GPL11154	Non-failing	heart
NF3	GSM1126614	Homo sapiens	GPL11154	Non-failing	heart
NF4	GSM1126615	Homo sapiens	GPL11154	Non-failing	heart
NF5	GSM1126616	Homo sapiens	GPL11154	Non-failing	heart
NF6	GSM1126617	Homo sapiens	GPL11154	Non-failing	heart
NF7	GSM1126618	Homo sapiens	GPL11154	Non-failing	heart
NF8	GSM1126619	Homo sapiens	GPL11154	Non-failing	heart
ICM1	GSM1126620	Homo sapiens	GPL11154	ischemic	heart
ICM2	GSM1126621	Homo sapiens	GPL11154	ischemic	heart
ICM3	GSM1126622	Homo sapiens	GPL11154	ischemic	heart
ICM4	GSM1126623	Homo sapiens	GPL11154	ischemic	heart
ICM5	GSM1126624	Homo sapiens	GPL11154	ischemic	heart
ICM6	GSM1126625	Homo sapiens	GPL11154	ischemic	heart
ICM7	GSM1126626	Homo sapiens	GPL11154	ischemic	heart
ICM8	GSM1126627	Homo sapiens	GPL11154	ischemic	heart

Table S7 Primer information

Gene	Forward	Reverse
LINC00452	5'-GTTGGTTCAGTGGGGGCTAT-3'	5'-GACAGGCTTGTAACGGAGGT-3'
AC011483.1	5'-CAGAACTACGTCCACTGGTCC-3'	5'-GCCACATCCACGCCATGTT-3'
CCR7	5'-TGAGGTCACGGACGATTACAT-3'	5'-GTAGGCCCACGAAACAAATGAT-3'
IL17RB	5'-ATGTCGCTCGTGCTGCTAAG-3'	5'AGCCACATTGAACGGTCGG-3'
MICB	5'-TCTTCGTTACAACCTCATGGTG-3'	5'-TGGCTCGCAGTTTGAGGAAT-3'
